# The Validation and Further Development of the Multidimensional Cognitive Load Scale for Physical and Online Lectures (MCLS-POL)

**DOI:** 10.3389/fpsyg.2021.642084

**Published:** 2021-03-18

**Authors:** Martin S. Andersen, Guido Makransky

**Affiliations:** Department of Psychology, University of Copenhagen, Copenhagen, Denmark

**Keywords:** cognitive load, confirmatory factor analysis, item response theory, online lecture, Rasch measurement

## Abstract

Cognitive load theory (CLT) has been widely used to help understand the process of learning and to design teaching interventions. The Cognitive Load Scale (CLS) developed by Leppink and colleagues has emerged as one of the most validated and widely used self-report measures of intrinsic load (IL), extraneous load (EL), and germane load (GL). In this paper we investigated an expansion of the CLS by using a multidimensional conceptualization of the EL construct that is relevant for physical and online teaching environments. The Multidimensional Cognitive Load Scale for Physical and Online Lectures (MCLS-POL) goes beyond the CLS's operationalization of EL by expanding the EL component which originally included factors related to instructions/explanations with sub-dimensions including EL stemming from noises, and EL stemming from both media and devices within the environment. Through three studies, we investigated the reliability, and internal and external validity of the MCLS-POL using the Partial Credit Model, Confirmatory Factor Analysis, and differences between students either attending a lecture physically or online (Study 2 and 3). The results of Study 1 (*N* = 250) provide initial evidence for the validity and reliability of the MCLS-POL within a higher education sample, but also highlighted several potential improvements which could be made to the measure. These changes were made before re-evaluating the validity and reliability of the measure in a new sample of higher education psychology students (*N* = 140, Study 2), and psychological testing students (*N* = 119, Study 3). Together the studies provide evidence for a multidimensional conceptualization cognitive load and provide evidence of the validity, reliability, and sensitivity of the MCLS-POL and provide suggestions for future research directions.

## Introduction

Cognitive load theory (CLT) posits that the strain put on working memory by the learning content plays a key role in whether or not the student succeeds in learning (Sweller et al., 2011a). A fundamental assumption is that working memory is limited in terms of capacity, but long term memory has a much greater capacity as information is stored in schemas (Chi et al., 1982). Thus, working memory becomes a form of bottleneck that requires instructors to design learning content in a way that can maximize the amount of information that is stored in the long term memory.

Originally, the CLT assumed that cognitive load was a unidimensional construct pertaining only to the total capacity of working memory (Ayres, 2018), and there is still disagreement about how to conceptualize different types of cognitive load (Kalyuga, 2011 Tindall-Ford et al., 2019). However, three distinct types of cognitive load are primarily described, including: Intrinsic Load (IL), which relates to the students perceived difficulty of the learning material, the difficulty of the learning material varies based on the materials composition and the materials' element interactivity (Sweller et al., 1998; Tindall-Ford et al., 2019). Extraneous Load (EL) which consist of non-intrinsic parts of the learning situation i.e., non-relevant information presented together with relevant information or inefficient instructional design, which will unnecessarily strain the working memory of the student (Sweller et al., 2011b). Finally, Germane Load (GL) is already existing cognitive resource which can ease the learning e.g., strategies for learning (Sweller et al., 2011b; Ayres, 2018). Some researchers have argued that GL is part of IL (Sweller, 2010; Kalyuga, 2011). Others argue that it makes sense to separate GL from IL and describe how GL is tied to actual effort that leads to a better understanding of the content (e.g., Klepsch et al., 2017), Finally, a recent article by Klepsch and Seufert argues that IL stems from a passive experience of a task, opposite GL that stems from an active experience of a task (Klepsch and Seufert, 2021).

Many attempts at measuring cognitive load have been proposed including objective tasks such as secondary tasks (Sweller et al., 2011c) and psychophysiological measures such as eye tracking (Zheng and Cook, 2012; Scharinger et al., 2020), and EEG (Antonenko et al., 2010; Makransky et al., 2019a; Baceviciute et al., 2020). Recently an article by Minkley, Xu, and Krell have compared subjective and objective factors of CL which found heart rate to be related to self-reported metal effort but not self-reported mental load, and self-reported mental effort and mental load predicted task performance better than heart rate measures (Minkley et al., 2021). However, the most common way to measure cognitive load is through self-report measures.

Previously, a single item measure by Paas (1992) has been widely used and further developed to measure several types of cognitive load (Ayres, 2006; Cierniak et al., 2009). However, single item scales have also been criticized due to several limitations including being too simplistic, making it difficult for learners to make sensible distinctions between the complexity of the material (IL) and inadequate instructions (EL; Kirschner et al., 2011). Several other self-report scales assess cognitive load with multiple items including a scale developed by Klepsch et al. (2017) which assess IL, EL, and GL, a measure to assess mental load and mental effort developed by Krell (2017), in addition to the cognitive load scale by Leppink et al. (2013), which we use in this article. We have chosen to build on the cognitive load scale by Leppink and colleagues as the items assess a broader domain such as a lecture.

In this article we aim to validate a revised version of Leppink and colleagues' Cognitive Load Scale (Leppink et al., 2013; CLS). The CLS has been widely used in educational settings, and several studies provide support for the validity and reliability of the instrument (e.g., Leppink et al., 2013; Hadie and Yusoff, 2016; Andersen and Makransky, 2020). This includes construct validity assessed through exploratory factor analysis (Leppink et al., 2013) or confirmatory factor analyses (Leppink et al., 2013; Hadie and Yusoff, 2016; Andersen and Makransky, 2020) and item response theory (Andersen and Makransky, 2020). The reliability has also been examined, typically through Cronbach's Alpha (Cronbach, 1951) or similar estimates (Leppink et al., 2013; Hadie and Yusoff, 2016; Andersen and Makransky, 2020) and furthermore the external validity has been examined by investigating how the scales are correlated to learning outcomes (Andersen and Makransky, 2020). Although there is mounting evidence of the reliability and construct validity of the CLS, there are still several gaps in this literature. A main gap in the literature are that there may be a need to revisit the content validity of the EL dimension of the CLS, and there is a need to evaluate the sensitivity of these potential dimensions of EL in physical and online lectures.

Regarding the content validity of the EL dimension, a recent study suggests that the EL may be a multidimensional construct consisting of several sub-components. Andersen and Makransky (2020) provide reliability and validity evidence that the EL dimension should be split into three subscales measuring distinct forms of EL in virtual reality environments. The subscales included: EL stemming from instructions (e.g., “The instructions and/or explanations used in the simulation were very unclear”), EL stemming from interaction (e.g., “The interaction technique used in the simulation made it harder to learn”), and EL stemming from the environment (e.g., “The virtual environment was full of irrelevant content”). This multidimensional conceptualization was theorized within immersive environments, but has not been suggested or investigated in traditional teaching environments. In this article we propose that the multidimensional conceptualization of EL is not only relevant in virtual learning environments, but rather that it is also necessary for accurately measuring cognitive load in physical and online lectures. Although, cognitive load theory does not clearly address the idea that disturbances and noises might increase EL (Sweller et al., 2011b), research suggests that multitasking using mobile devices reduce learning (Kuznekoff and Titsworth, 2013; Chen and Yan, 2016). Furthermore, research suggests that noises in learning environments can also influence learning (Ali, 2013; Servilha and Delatti, 2014), thus the idea that noises and disturbances add to EL seems straightforward. Therefore, we have devised items for three subscales to measure EL in relation to physical and online lectures addressing contemporary issues, including noises in the environment or distractions from devices such as mobile phones, which might provoke EL. In addition to the original conceptualization of EL from Leppink et al. (2013) that includes instructions and or explanations (e.g., “*The instructions and/or explanations during the activity were very unclear*”), our theoretical conceptualization of EL includes sub-dimensions stemming from noise (e.g., “*Noises in the environment made it difficult to focus on the learning content*”), and devices (e.g., “*My activities on my phone/computer made it difficult to focus on the learning content*”). Besides the newly developed EL subscales we also employed the Intrinsic Load subscale (e.g., “*The topics covered in the activity were very complex*”), and the Germane Load (GL) subscales (e.g., “*The activity really enhanced my understanding of the topic(s) covered*”). The new measure is labeled the Multidimensional Cognitive Load Scale for Physical and Online Lectures (MCLS-POL and can be seen in Table 1). Although several factors influence teaching in both online (Elkaseh et al., 2015) and offline learning (McKenzie and Schweitzer, 2001; Kappe and van der Flier, 2012) environments, we propose that these components of cognitive load are specifically relevant factors that can influence learning in offline (Klatte et al., 2013; Chen and Yan, 2016; Cerdan et al., 2018) and online lectures (Blasiman et al., 2018; Zureick et al., 2018; Costley et al., 2020). Specifically, with the global COVID-19 pandemic, the use of online teaching platforms is quickly increasing (König et al., 2020) and there is evidence that factors such as noise (Servilha and Delatti, 2014) and disturbances from devices (Chen and Yan, 2016) can create cognitive load when learning.

**Table 1:** Items and scales included in the Study 1.

**Scale**	
IL	The topics covered in the activity were very complex.
IL	The activity covered theories that I perceived as very complex.
IL	The activity covered concepts and definitions that I perceived as very complex.
EL ins	The instructions and/or explanations during the activity were very unclear.
EL ins	The instructions and/or explanations were, in terms of learning, very ineffective.
El ins	The instructions and/or explanations were full of unclear language.
EL noi	Other students talking in the classroom made it difficult to focus on the learning content.
EL noi	Students talking to me during the activity made learning ineffective.
EL noi	Other noises and distractions during the activity made it hard to learn.
EL dev	My activities on my phone/computer made it difficult to focus on the learning content.
EL dev	Messages and notifications from my phone/computer made learning unclear.
EL dev	Others' phone/computer use distracted me, making it hard to learn.
GL	The activity really enhanced my understanding of the topic(s) covered.
GL	The activity really enhanced my knowledge and understanding of cognitive load.
GL	The activity really enhanced my understanding of the theories covered.
GL	The activity really enhanced my understanding of concepts and definitions.

*IL, Intrinsic Load; EL Ins, Extraneous Load Instructions; EL Noi, Extraneous Load Noises; EL Dev, Extraneous Load Devices; GL, Germane Load*.

A related gap in the literature that we attempt to account for in this article is the limited number of studies that investigate the sensitivity of the different dimensions of cognitive load in realistic learning environments. Currently some studies have found meaningful differences of groups in cognitive load (Klepsch et al., 2017; Andersen and Makransky, 2020) and others have found predictive validity through regression analyses (Zukić et al., 2016; Andersen and Makransky, 2020).

In this paper we conduct three studies. In the first study, we validate each sub-scale of the MCLS-POL using the Partial Credit Model (PCM) from Item Response Theory (IRT), and second, we used confirmatory factor Analysis (CFA) to investigate the structural validity of the MCLS-POL. In the second and third studies we implement changes to the scale and investigate the sensitivity of the different sub-dimensions during a lecture in a higher education psychology bachelor course on the topic of educational psychology (Study 2), and a different lecture in a higher education psychology masters course on the topic of psychological testing (Study 3) which both took place in the Fall 2020 semester. Importantly, the setting of Study 2 and Study 3 took place during the COVID-19 pandemic and students were selected to either attend the lecture in person or online via Zoom (Kohnke and Moorhouse, 2020) which gave us the opportunity to investigate if the components of cognitive load differed across settings. Finally, we compared scores across Study 2 and Study 3 to investigate whether the scales would reflect the difference between the two courses. Thus, in this article our aim is to investigate whether it is possible to develop and validate questionnaires measuring cognitive load, particularly the expanded scales of extraneous cognitive load pertaining to EL from instruction, noise, and devices. In regard to comparing online with off-line learning, our research hypotheses are that there should be no differences in terms of intrinsic cognitive load or germane cognitive load between online and offline lectures as the materials and the possible germane resources should be similar. Furthermore, we don't expect any differences in relation to EL from instructions, since students in both online and off-line learning environments receive the same instructions. However, we expected to find differences across the newly developed extraneous cognitive load scales related to devices and noises because there will be differences between the online and off-line learning contexts which could influence these factors of EL.

## Study 1

### Methods Study 1

#### Sample

Data was collected at a European University during the fall 2019 semester. The psychology students (*N* = 250) were asked to voluntarily answer a short online survey in relation to their current course in educational psychology (*n* = 120) or psychological testing (*n* = 130). A total of 80.8% reported being females (*n* = 202), 18.4% males (*n* = 46), and 0.8% (*n* = 2) reported another gender than male or female. The mean age was 25.46 with an *SD* of 5.45.

#### Item Development

A team of subject matter experts consisting of an expert in educational psychology, a specialist in human computer interaction, and a psychometrician further developed the scales of Leppink and colleagues' CLS. Based on a previous study where the EL scale was conceptualized using three separate EL subscales aimed at measuring CL in virtual reality (Andersen and Makransky, 2020), we took a similar approach by conceptualizing EL as a multidimensional construct with several subscales. However, instead of being aimed at learning in virtual reality it was aimed at learning during lectures, and was based on the literature that specified the factors that could create extraneous cognitive load within physical and online lectures. We aimed to make the items so generic that it should be possible to transfer them from one context to another without rewriting them, however in keeping with Leppink et al. (2013) formulation item 2 of the Germane Load scale specifically mentions the course subject (i.e., “The activity really enhanced my knowledge and understanding of [course subject].”) and therefore has to be modified accordingly for each study (see Table 1 for all items used in Study 1).

#### Statistical Analyses

In this study, we employ two methodologies to investigate the construct validity of the MCLS-POL. The first methodology is that of item response theory (IRT; Embretson and Reise, 2000) which estimates a probability function for endorsing each item of a scale in relation to the scales' total score, that allows for detailed analyses of each item. The second methodology is that of confirmatory factor analysis (CFA; Kline, 2011), in which we model the relationship between items and several latent variables called factors. Therefore, we can evaluate the fit of a model including all items and all scales as latent factors and their relation in just one model.

As the IRT approach is focused on each individual scale, it makes sense to conduct the IRT analyses first and let the knowledge from the IRT analyses inform the overall CFA model which will contain all scales. For an IRT model of the Rasch model family to be valid it must live up to five assumption (Rosenbaum, 1989). The assumptions are: (a) unidimensionality; the scale must measure one latent construct only, (b) the items must be monotonic in relation to the total scale, (c) the items must be locally independent, i.e., the items are conditionally independent after accounting for the total score, (d) the items must not show differential item functioning, e.g., students of the same ability should have equal probability of endorsing an item regardless of gender or age, (e) items must be homogenous such that that the rank order of the items of the difficulties remain the same despite differing abilities of the respondent, e.g., the most difficult item should be the most difficult item to endorse for all respondents. We will address each assumption for every scale in the analyses.

In some cases where we find deviations from assumptions of no Differential Item Functioning (DIF) (d) or no Local Dependence (LD) (c), we are still able to obtain close to optimal measurement. When DIF or LD is uniform, we can model this with a graphical log linear Rasch model (GLLRM; Kreiner and Christensen, 2004). This model can account for the differences in item functioning when DIF is present, however when using sum scores we will need to equate across DIF affected groups to make the sums scores comparable. When uniform LD is present it does not influence the sums scores, however LD dependency will inflate estimates of reliability such as Cronbach's Alpha (Cronbach, 1951) and we will instead use a Monte Carlo method to compute the estimate of reliability (Hamon and Mesbah, 2002). Factor analysis can be used to create a model where each item's relation to the scales is part of a matrix of regressions. In the confirmatory approach, we restrict the model, such that items of a given scale only load on the hypothesized factor and not any of the other factors. This allow us to not only consider the properties of the scales independently as in IRT, but also to investigate if there might be overlap between items across and other scales.

In Study 1 for IRT analyses of the polytomous items of the CL scales, we used the Partial Credit Model (PCM; Masters, 1982) in the Digram program (Kreiner and Nielsen, 2013). An overall test of DIF and homogeneity was conducted with Andersen's conditional likelihood test (Andersen, 1973). Item fit was assessed with item rest score correlations (Christensen and Kreiner, 2013). For the analyses of items-wise DIF in relation to gender, age (grouped by 1 = 0–23, and 2 = 23 and above), and course and LD we used Keldermans' likelihood ration test (Kelderman, 1984) and Goodmann and Kruskal's partial gamma correlation (Kreiner and Christensen, 2004).

For Pure PCM models in Study 1 we used Cronbach's Alpha (Cronbach, 1951) to estimate reliability. For scales with evidence of LD we used a Monte Carlo procedure to estimate the reliability since Cronbach's Alpha is prone to inflation for scales with local dependence. To account for false discovery rates due to the multiple testing we used the Benjamini and Hochberg procedure (Benjamini and Hochberg, 1995). For example, we employed the procedure to test of all possible item pairs in relation to local dependence.

To conduct the CFA we used the Lavaan package (version 0.6-5) in the R statistical programming language (version 3.6.3). To estimate the loading of the model, we used the diagonally least square method (Li, 2016), since the items were ordinal. We used the Comparative Fit Index (CFI) and the Tucker Lewis Index (TLI) with values above 0.95 to indicate acceptable fit (Hu and Bentler, 1999). Besides CFI and TLI we used the Root Mean Square Error of Approximation (RMSEA) and the Standardized Root Mean Square Residual (SRMR) were values below 0.06 and 0.08, indicate a good fit, respectively (Hu and Bentler, 1999).

### Results Study 1

#### Results of Fit to the Partial Credit Model

The Rasch analysis of the IL scale indicated no evidence against the fit to a PCM. The overall test found no evidence of breach of homogeneity, the overall test, or the item-wise tests of DIF in relation to gender, age, or course. There was no evidence against item fit and no evidence of local dependence (see Table 2). The reliability of the scale measured in terms of the Cronbach's Alpha was 0.89. Therefore, we concluded that the scale provided valid and reliable measurement.

**Table 2:** Results for the Rasch analyses of the scales in Study 1.

**Scale**	**Overall test**	**Item fit**	**DIF gender**	**DIF age**	**DIF course**	**LD**	***r***
IL	✓	✓	✓	✓	✓	✓	0.89
EL Ins	✓	✓	✓	✓	%	✓	0.84
EL Noi	%	✓	%	%	%	%	0.81
EL Dev	✓	✓	✓	%	✓	%	0.62
GL	✓	✓	%	✓	✓	%^6^	0.90

*IL, Intrinsic Load; EL Ins, Extraneous Load Instructions; EL Noi, Extraneous Load Noises; EL Dev, Extraneous Load Devices; GL, Germane Load; DIF, Differential Item Functioning; LD, Local Dependence; r, reliability. %, unacceptable, where the check mark should be acceptable*.

The analyses of EL instructions scale exhibited evidence of DIF in relation to course for item 1, such that it was easier for students of psychological testing to endorse the statement “*The instructions and/or explanations during the activity were very unclear”* than for students of educational psychology, despite similar levels of EL related to instructions. When the DIF was added to a graphical log linear Rasch model, then neither the overall test nor the item-wise test showed evidence of DIF. There was no evidence of breach of homogeneity, or against item fit. Finally, there was no evidence of local dependence between items and the reliability was 0.84. Therefore, we concluded that the scale provided valid and reliable measurement.

The Analyses of the Extraneous load scale for noise, showed evidence of both DIF and local dependence. Although, no evidence against item fit and with a reliability coefficient of 0.81, it was not possible to find a working model with LD and DIF which could converge, thus there was no fit to a PCM of a GLLRM for the EL N scale.

For the extraneous load scale in relation to devices, we found evidence of DIF in relation to age for item 2, meaning it was easier for younger students to endorse the statement: “*Messages and notifications from my phone/computer made learning unclear.”* and local dependence between item 1 “*My activities on my phone/computer made it difficult to focus on the learning content,”* and item 2 “*Messages and notifications from my phone/computer made learning unclear*.” After adding these two deviations from the PCM to a GLLRM, there was no further evidence of DIF or LD, and no evidence against item fit or homogeneity, but the reliability of the scale was only 0.62. Therefore, we conclude that the scale did not fit the PCM, and that we were able to model the DIF and LD, however, the scale had low reliability.

To achieve a working model for the Germane Load scale item 2: “*The activity really enhanced my knowledge and understanding of cognitive load/psychological testing.”* was omitted. Further analysis of the remaining three items showed evidence of DIF relative to gender for item 4, Such that it was easier for females to endorse: “*The activity really enhanced my understanding of concepts and definitions,”* despite having the same level of germane load. Furthermore, we found evidence of local dependence between item 3: “*The activity really enhanced my understanding of the theories covered.”* and item 4: “*The activity really enhanced my understanding of concepts and definitions.”* After these two instances were added to a GLLRM, there was no further evidence of DIF or LD and no evidence against item fit or against homogeneity, and the scale had a reliability of 0.90. Therefore, we concluded that the scale did not fit a pure Partial Credit Model, but could still provide close to optimal measurement after accounting for DIF and LD.

#### Results of Fit to the Confirmatory Factor Analysis

A Confirmatory Factor analysis was run including all items from all scales with the exception of item 2 from the GL scale because it did not fit the Partial Credit model in the previous analysis, and without the EL Noise scale which did not converge during the PCM analyses. The model grouped each scale's item so they formed a latent construct for each scale, e.g., all IL items loading only on the latent construct of IL. The model achieved acceptable fit values. The CFI was 0.999 and the TLI was 0.999. The RMSEA was <0.001 and SRMR was 0.041, thus all values indicated the model was acceptable.

### Discussion Study 1

Overall the IRT and CFA analyses provided positive evidence of the construct validity of the MCLS-POL with few minor cases of LD and DIF which could be modeled. However, three major issues were identified. The first was that the EL Noise scale would not converge to a meaningful model. Adding instances of local dependence to the model led to other instances other local dependence until the program could no longer converge. Second, although the EL devices scales converged to a model after accounting for LD between two items and accounting for DIF, the scales reliability was lower than conventional cut-off for satisfactory reliability. Finally, item 2 from the GL scale had to be eliminated as it did not fit the model. These issues were dealt with in a revision of the MCLS-POL which is described in Study 2. Overall we found evidence against the validity of the EL noise scale, but we found no evidence against the validity of the other scales.

## Study 2

Study 2 was conducted to improve the MCLS-POL based on the results of Study 1. We were interested in investigating the criterion validity of the different sub-scales within the MCLS-POL in addition to testing the reliability and validity of the measure using the PCM and CFA as in Study 1. Sensitivity was tested by using an experimental design where students experienced a lecture in educational psychology either physically or online through Zoom. An experiment was possible because restrictions due to the COVID-19 pandemic meant that approximately half of the students were assigned to a group who had to follow the lecture online instead of physically in order to increase physical distancing in the lecture hall. The students attending the lecture online followed the same lecture as the students who were physically present, while online the students could choose between seeing just the lecture slides or the teacher in front of the lecture slides, while listening to teacher speak. To examine if the uses of scales scores made sense we used the validity frame work of Kane (2013), and examined whether the scales showed meaningful differences such that online students experienced more EL than off-line students as hypothesized in the introduction.

### Item Revision

Before conducting the study, we reformulated the wording of the items for the EL Noise scale so the item content became more general based on the finding that the scale did not fit the PCM in Study 1. For example, we changed the wording of item 1 from “*Other students talking in the classroom made it difficult to focus on the learning content*” to “*Noises in the environment made it difficult to focus on the learning content*” (see Table 3 for all items used in Study 2 and Study 3). This was also useful as restrictions due to the COVID-19 pandemic meant that approximately half of the students were assigned to a group who had to follow the lecture online to increase physical distancing in the lecture hall. Given the difference between participating in a lecture physically and online we expected differences in the EL sub-scales of Noise, and Devices. We also added two more items to the EL Devices sub-scale as the results from Study 1 indicated that the scale had a low reliability.

**Table 3:** Items and scales included in the Study 2 and Study 3.

**Scale**	
IL	The topics covered in the activity were very complex.
IL	The activity covered theories that I perceived as very complex.
IL	The activity covered concepts and definitions that I perceived as very complex.
EL Ins	The instructions and/or explanations during the activity were very unclear.
EL Ins	The instructions and/or explanations were, in terms of learning, very ineffective.
EL Ins	The instructions and/or explanations were full of unclear language.
EL Ins	Low quality audio made the instructions hard to follow.
EL Noi	Noises in the environment made it difficult to focus on the learning content.
EL Noi	Distractions in the environment made learning ineffective.
EL Noi	Unrelated events occurring in the environment made it difficult to focus.
EL Dev^a^	My activities on my phone/computer made it difficult to focus on the learning content.
EL Dev^a^	Messages and notifications from my phone/computer made learning unclear.
EL Dev^b^	Others' phone/computer use distracted me, making it hard to learn.
EL Dev	Technical issues made learning ineffective.
EL Dev	Problems with technology made it difficult to focus.
GL	The activity really enhanced my understanding of the topic(s) covered.
GL	The activity really enhanced my knowledge and understanding of [course subject].
GL	The activity really enhanced my understanding of the theories covered.
GL	The activity really enhanced my understanding of concepts and definitions.

*IL, Intrinsic Load; EL Ins, Extraneous Load Instructions; EL Noi, Extraneous Load Noises; EL Dev, Extraneous Load Devices; GL, Germane Load*.

a *Combined to make an EL Media scale*.

b *Omitted from final analyses*.

### Sample

Data were collected at a European University during the fall 2020 semester. The psychology students (*N* = 140) were asked to voluntarily answer a short online survey in relation to their current course in educational psychology. A total of 76.4% reported being females (*n* = 107), 22.9% males (*n* = 32), and 0.7% (*n* = 1) did not wish to answer the question. The mean age was 23.29 with a *SD* of 3.83. Due to the COVID-19 pandemic, the University restricted the number of students who could attend the lecture physically and students were assigned to either attend in person or online through Zoom prior to the lecture. A total of 62 students attended the lecture in person, the rest of the students attended the lecture through the Zoom online streaming service (*n* = 78). The students experienced the same lecture with the only difference being their presence in the classroom, or experiencing it online through Zoom. The MCLS-POL was administered at the end of the lecture through SurveyMonkey.

### Statistical Analyses

In Study 2 for IRT analyses of the polytomous items of the CL scales, we used the Partial Credit Model (PCM; Masters, 1982) in RUMM (Andrich et al., 2003), the switch from Digram to RUMM was made as RUMM is able to handle scales based on only two items which became a necessity in Study 2. An overall test of fit to the PCM was conducted with a chi-square test, where significance indicate misfit in relation to the model (Pallant and Tennant, 2007). Item fit was deemed acceptable if the residuals of the models were within −2.5 and +2.5 (Pallant and Tennant, 2007). Local dependence was assessed by examining the residual correlations between items, where we expected the residual correlation to be close to zero. We used items residuals above 0.20 as indicative of local dependence (Christensen et al., 2017). The presence of DIF was examined through analysis of variance in items scores across age, gender, and whether the student was present physically or attended the lecture online, in cases where we tested with multiple items we corrected the *p*-values with the Bonferroni correction to adjust for false discovery rates.

### Results for Study 2

#### Results of Fit to the Partial Credit Model

The Rasch analyses of the five scales provide almost no evidence against fit in the overall test or in relation to item fit. A minor deviation was found for the IL scale as the overall test rejected at fit (*p* < 0.001) however this might be due to item 2 which fit to the PCM was rejected at *p* > 0.05 but not *p* > 0.01 after Bonferroni correction. Furthermore, the residuals for the item fit was between −2.5 and 2.5, thus we concluded the scale fit.

The only major deviation from the model was related to the EL Devices scale where we identified strong evidence of multidimensionality. After reexamining the wording of the items it was clear that the items were assessing two separate constructs: One measuring the EL from Media with the following items (item 1, “*My activities on my phone/computer made it difficult to focus on the learning content”* and item 2, “*Messages and notifications from my phone/computer made learning unclear”*) and the second measuring EL from devices with the following items (item 4, “*Technical issues made learning ineffective*” and item 5, “*Problems with technology made it difficult to focus*”). Furthermore, item 3 did not fit any of the scales and was eliminated. After the split both scales fit the model and for all scales the reliability was satisfactory (see **Table 6**).

For the other sub-scales, we only found evidence of one instance of DIF depending on whether the students attended the course physically or online in two items on the EL Instructions scale, such that is was easier for physically present students to endorse the statement in item 2 “*The instructions and/or explanations were, in terms of learning, very ineffective*” than the students attending the lecture online. Opposite of this it was easier for the online students to endorse the statement of item 4 “*Low quality audio made the instructions hard to follow*,” than for the physically present students, despite having similar levels of EL in relation to instructions. For the GL scale we again omitted item 2 to achieve a working model, the *p*-value for item fit of item 3 in the GL scale was 0.0163 (Bonferroni corrected cut-off was 0.016). However, as the residuals was inside −2.5 to + 2.5 we accepted it. Table 4 illustrates how all other items fit the PCM providing evidence of the validity and reliability of the revised version of the MCLS-POL.

**Table 4:** Results for the Rasch analyses of the scales in Study 2 in RUMM.

**Scale**	**Overall test**	**Item fit**	**DIF gender**	**DIF age**	**DIF location**	**LD**	***r***
IL	%	✓	✓	✓	✓	✓	0.86
EL Ins	✓	✓	✓	✓	%	✓	0.73
EL Noi	✓	✓	✓	✓	✓	✓	0.85
EL Med	✓	✓	✓	✓	✓	✓	0.85
EL Dev	✓	✓	✓	✓	✓	✓	0.87
GL	✓	✓	✓	✓	✓	✓	0.88

*IL, Intrinsic Load; EL Ins, Extraneous Load Instructions; EL Noi, Extraneous Load noise; EL Med, Extraneous Load Media; EL Dev, Extraneous Load devices; GL, Germane Load; DIF, Differential Item Functioning; LD, Local Dependence; r, reliability. %, unacceptable, where the check mark should be acceptable*.

#### External Validity Results

Table 5 shows the difference between being physically present and attending the lecture online. Independent samples *t*-tests were conducted to investigate if the differences between the physical and online groups were significant. For the IL scale there was no significant difference between being physically present or attend online [*t*_(138)_ = −1.281, *p* = 0.202] as expected. Furthermore, EL Noise was significantly higher [*t*_(138)_ = −5.795, *p* < 0.001] for online students (*M* =2.368, *SD* = 0.962) than for physically present students (*M* = 1.591, *SD* = 0.614). EL Media was significantly higher [*t*_(138)_ = −3.401, *p* < 0.001] for online students (*M* =2.820, *SD* =1.165) than for physically present students (*M* = 2.226, *SD* = 0.904). EL Devices was significantly higher [*t*_(138)_ = −3.290, *p* < 0.001] for online student (*M* = 2.532, *SD* = 1.070) than for physically present students (*M* = 2.016, *SD* = *0*.784). All of these fit the a-priori hypotheses. However, contrary to the a-priori predictions EL Instructions was significantly higher [*t*_(138)_ = −4.558, *p* < 0.001] for online student (*M* = 2.234, *SD* = 0.718) than for physically present students (*M* =1.762, *SD* = 0.505). The online students (*M* = 3.724, *SD* = 0.713) also experienced significantly [*t*_(138)_ = 2.071, *p* = 0.040] lower GL than the physically present students (*M* = 3.944, *SD* = 0.538). These results suggest that the MCLS-POL is sensitive to differences between students learning in different environments and provides support for the external validity of the measure. However, students also reported different levels of EL related to instructions and GL which was not expected in the a-priori predictions.

**Table 5:** A comparison of the students who attended the course online and those who were physically present on the scales in the MCLS-POL in Study 2.

**Scale**	**Online**		**Physical present**		***t*_(138)_**	***p***	**Cohen' s *d***
	***M***	***SD***	***M***	***SD***			
IL	2.773	0.717	2.586	0.731	−1.281	0.202	0.259
EL Ins	2.234	0.718	1.762	0.505	−4.558	<0.001	0.746
EL Noi	2.368	0.962	1.591	0.614	−5.795	<0.001	0.940
EL Med	2.820	1.165	2.226	0.904	−3.401	<0.001	0.746
EL Dev	2.532	1.070	2.016	0.784	−3.290	<0.001	0.541
GL	3.724	0.713	3.944	0.538	2.071	0.040	0.343

*IL, Intrinsic Load; EL Ins, Extraneous Load instructions; EL Noi, Extraneous Load Noises; EL Med, Extraneous Load Media; EL Dev, Extraneous Load Devices; GL, Germane*.

### Discussion Study 2

Study 2 revealed that the EL Devices scale should be split into two sub-scales in order to create valid measurement. A meaningful categorization was made by creating an EL Media subscale, and an EL Devices subscale. The EL Media sub-scale consisted of the item “*My activities on my phone/computer made it difficult to focus on the learning content*” and the item “*Messages and notifications from my phone/computer made learning unclear*.” The EL Devices sub-scale consisted of the item ”*Technical issues made learning ineffective*” and the item “*Problems with technology made it difficult to focus*.” Due to the item wording (i.e., the first two items pertaining to disturbances from the devices and the second two items pertaining to technology in general) it made sense to split the scale into two distinct scales. Furthermore, comparing the students based on whether they were physically present or attending the lecture online revealed meaningful differences such that the students attending the lecture online experience significantly more EL related to instructions, noise, media, and devices, as well as significantly less GL than the students who were physically present in the lecture hall. The difference between the groups on IL was not significant. To investigate if these results would replicate in a new setting we conducted a follow-up study.

## Study 3

Study 3 was conducted to test the validity of the MCLS-POL in a new context by replicating Study 2 in a sample of psychology master students who were participating in a lecture about psychological testing. The same statistical analyses were conducted as in Study 2.

### Sample

Data was collected at a European University during the fall 2020 semester. The psychology master students (*N* = 119) were asked to voluntarily answer a short online survey in relation to a course in psychological testing. A total of 89.1% reported being females (*n* = 106), and 10.9% males (*n* = 13). The mean age was 27.25 with an *SD* of 6.65. Similar to Study 2, students were assigned to attending the course physically or online prior to the lecture but students who were allowed to attend physically were given the option of attending online. A total of 27 students attended the lecture in person, the rest of the students attended the lecture through the Zoom online streaming service (*n* = 92). The students attending in person had to wear a mask while entering the lecture hall, which they could remove while seated and they had to sit with distance between them. The teacher also had to wear a mask when entering the lecture hall, but the teacher was allowed to remove the mask during the lecture.

### Results for Study 3

#### Results of fit to the Partial Credit Model

The Rasch analyses of the six scales provide almost no evidence against fit in the overall test or in relation to item fit. We only found evidence DIF depending on whether the students attended the course online or by being physically present in relation to two items on the EL Noise scale, such that it was easier for physically present students to endorse “*Noises in the environment made it difficult to focus on the learning content*,” while it was easier for student attending online to endorse “*Distractions in the environment made learning ineffective*,” despite having the same level of EL related to noise. Again we split the EL device scale into two separate two-item scales. One measuring the EL from media and the second measuring EL from devices. After the split both scales fit the model and for all scales the reliability was above 0.80 and thus satisfactory (see Table 6).

**Table 6:** Results for the Rasch analyses of the scales in Study 3 RUMM.

**Scale**	**Overall test**	**Item fit**	**DIF gender**	**DIF age**	**DIF zoom**	**LD**	***R***
IL	✓	✓	✓	✓	✓	✓	0.93
EL Ins	✓	✓	✓	✓	✓	✓	0.84
EL Noi	✓	✓	✓	✓	%	✓	0.81
EL Med	✓	✓	✓	✓	✓	✓	0.85
EL Dev	✓	✓	✓	✓	✓	✓	0.90
GL	✓	✓	✓	✓	✓	✓	0.90

*IL, Intrinsic Load; EL Ins, Extraneous Load Instructions; EL Noi, Extraneous Load noise; EL Med, Extraneous Load Media EL Dev, Extraneous Load Devices; GL, Germane Load; DIF, Differential Item Functioning; LD, Local Dependence; r, reliability. %, unacceptable, where the check mark should be acceptable*.

#### External Validity Results

Table 7 shows the difference between being physically present and attending the lecture online. The EL noise and the EL devices scales showed significant differences across type of attendance as predicted. For the EL Noises scale scales the students who attended the lecture online (*M* = 2.370, *SD* = 0.745) experienced significantly [*t*_(117)_ = −3.878, *p* < 0.001] more EL related to noises than the students who were physically present (*M* = 1.741, *SD* = 0.669). Similarly, on the EL Devices scale the students who attended the lecture online (*M* = 2.179, *SD* = 1.015) experienced significantly [*t*_(117)_ = −2.076, *p* = 0.040] more EL related to devices than the students who were physically present (*M* = 1.740, *SD* = 0.764). However, although the online lecture group (*M* = 2.696, *SD* = 1.021) also experienced more EL related to media than the students who were physically present (*M* = 2.333, *SD* = 1.047) this difference did not reach statistical significance [*t*_(117)_ = −1.608, *p* = 0.111]. Finally, the difference between the groups on IL, EL related to instructions, and GL were not statistically significant as predicted. The results suggest that the EL noise and EL devices scales within the MCLS-POL is sensitive to differences between students learning in different environments and provides support for the external validity of the measure.

**Table 7:** A comparison of the students who attended the course online and those who were physically present on the scales in the MCLS-POL in Study 3.

**Scale**	**Online**		**Physical present**		***t*_(117)_**	***p***	**Cohen' s *d***
	***M***	***SD***	***M***	***SD***			
IL	3.181	0.836	3.200	0.718	0.092	0.927	0.023
EL Ins	2.544	0.745	2.463	0.814	−0.848	0.629	0.106
EL Noi	2.370	0.950	1.741	0.669	−3.878	<0.001	0.703
EL Med	2.696	1.021	2.333	1.047	−1.608	0.111	0.354
EL Dev	2.179	1.015	1.740	0.764	−2.076	0.040	0.455
GL	3.266	0.677	3.037	0.822	−1.471	0.144	0.322

*IL, Intrinsic Load; EL Ins, Extraneous Load Instructions; EL Noi, Extraneous Load Noises; EL Med, Extraneous Load Media; EL Dev, Extraneous Load Devices; GL, Germane Load*.

### Discussion Study 3

Study 3 showed that all six scales provide valid measurement. Furthermore, comparing the students based on whether they were physically present or attending the lecture online revealed differences in a way that the students attending the lecture online experience significantly more EL related to noises and devices than the students who were physically present, other than those two scales there were no significant difference in the experienced CL. These results suggest that it is important to have a multidimensional conceptualization of EL as different components of EL can influence learning in physical and online environments differently.

It is not immediately clear why the differences in mean between students attending the lecture physically and students attending online in Study 3 are not similar to that of students in Study 2. One explanation might be that the master students attending Psychological Testing were more accustomed to lectures than the bachelor students attending Educational Psychology. Thus, we might reason that more experienced learners are less hampered by different types of extraneous load despite attending lectures online.

## Results of Combining Data From Study 2 and Study 3

### Results of the Confirmatory Factor Analysis

Before comparing the sum scores of Study 2 and Study 3 we conducted a confirmatory factor analysis with all items loading on their respective factors by combining data from Study 2 and Study 3. The fit indices were CFI = 0.99, TLI = 0.99, and both RMSEA and SRMR = 0.06 which are all satisfactory for the six factor model.

### Comparing Cognitive Load Across Study 2 and Study 3

Although the samples of psychology students in Study 2 and Study 3 differed because the students in the educational psychology course were second year bachelor students and the students in the psychological testing course were master students, we were interested in comparing the cognitive load ratings across the studies. Since psychological testing is considered a more cognitive straining course by many students, we wanted to compare the scales across the two courses. To ensure the scales were comparable across the studies we performed a CFA which yielded satisfactory fit indices. When comparing across courses we found IL to be significantly [*t*_(257)_ = −5.317, *p* < 0.001] higher for students from psychological testing (*M* = 3.18, *SD* = 0.81) than for students of educational psychology(*M* = 2.67, *SD* = 0.72). Similarly, We found EL instruction to be significantly [*t*_(257)_ = −5.625, *p* < 0.001] higher for students from psychological testing (*M* = 2.53, *SD* = 0.76) than for students of educational psychology (*M* = 2.03, *SD* = 0.67). On the other hand GL was significantly [*t*_(257)_ = 7.158, *p* < 0.001] lower for students of psychological testing(*M* = 3.21, *SD* = 0.65) than for students of educational psychology (*M* = 3.82, *SD* = 0.65, see Table 8). The differences between the groups were not significant for the other scales.

**Table 8:** Difference between scores on the MCLS-POL in Study 2 and Study 3.

**Scale/course**	**Educational psychology**		**Psychological testing**		***t***	***p***	**Cohen' s *d***
	***M***	***SD***	***M***	***SD***			
IL	2.67	0.72	3.18	0.81	−5.317	<0.001	0.67
EL Ins	2.03	0.67	2.53	0.76	−5.625	<0.001	0.70
EL Noi	2.02	0.91	2.23	0.93	−1.775	0.077	0.23
EL Med	2.56	1.09	2.61	1.04	−0.423	0.673	0.05
EL Dev	2.30	0.98	2.08	0.98	1.827	0.069	0.22
GL	3.82	0.65	3.21	0.72	7.158	<0.001	0.89

*IL, Intrinsic Load; EL Ins, Extraneous Load Instructions; EL Noi, Extraneous Load Noises; EL Med, Extraneous Load Media; EL Dev, Extraneous Load Devices; GL, Germane Load*.

## Conclusion

Through three studies we describe the further development and validation of the MCLS-POL. We provide evidence of the validity and reliability of the expanded CLS which supports the multidimensional conceptualization of cognitive load. Overall there was evidence of meaningful external validity in terms of meaningful group difference between students experiencing a lecture physically and students who experience the same lecture online. However, since one of the scale was split into two separate scale with only two items each, we highly recommend that researchers wishing to use these scale enhance these two scales by adding more items to them.

The studies also reveals meaningful challenges that students as well as lectures face as more teaching is conducted online. The results from Study 2 revealed that the students attending the lectures online experienced more EL on all four EL load sub-scales, meaning that they experienced extraneous cognitive load related to instructions, noises, media, and devices, which was greater than what the students who were physically present experienced. Furthermore, the physically present students in Study 2 also reported higher GL than the students who attended the lecture online.

The students attending the lecture online in Study 3 similarly experienced more cognitive load related to EL from media and devices than the physically present students. The finding that there were no differences on IL between the students who experienced the lecture physically or online in Studies 2 and 3, but there were differences in different components of EL suggests that the MCLS-POL is sensitive at identifying different components of cognitive load.

A limitation of these studies is the lack of measurement of learning, and the subsequent hypothetical analyses of differences in learning across the students attending either online or off-line and correlations between the CL scales and learning outcome. However, as we examined the cognitive load across differing course, creating a measure of learning with similar properties across courses was difficult. Future studies might address this by examining learning in just one type of course.

The reason for providing online lectures in Studies 2 and 3 was due to the extraordinary consequences of the COVID-19 pandemic in 2020. However, many universities are aiming at providing more online lectures, due to several advantages such as accessibility issues e.g., the ability to reach more students and allow students to access high quality educational opportunities even though they are unable to be physically present (Waschull, 2001; Cascaval et al., 2008; French and Kennedy, 2017; Makransky et al., 2019b). In contrast to the benefits this article highlights some of the caveats of online teaching related to the strain it can provide for students in terms of more EL, which should be addressed when conducting online lectures.

## Data Availability Statement

The raw data supporting the conclusions of this article will be made available by the authors, without undue reservation.

## Ethics Statement

Ethical review and approval was not required for the study on human participants in accordance with the local legislation and institutional requirements. The patients/participants provided their written informed consent to participate in this study.

## Author Contributions

MA collected data conducted analyses and wrote the article. GM collected data and supervised the analyses and the writing. All authors contributed to the article and approved the submitted version.

## Conflict of Interest

The authors declare that the research was conducted in the absence of any commercial or financial relationships that could be construed as a potential conflict of interest.
